# Blockade of RBP-J-Mediated Notch Signaling Pathway Exacerbates Cardiac Remodeling after Infarction by Increasing Apoptosis in Mice

**DOI:** 10.1155/2018/5207031

**Published:** 2018-07-03

**Authors:** Yanru He, Si Pang, Jia Huang, Kongbo Zhu, Jiayi Tong, Yaoliang Tang, Genshan Ma, Lijuan Chen

**Affiliations:** ^1^Department of Cardiology, Zhongda Hospital Affiliated to Southeast University, Nanjing, China; ^2^Medical School of Southeast University, Nanjing, China; ^3^Department of Cardiovascular Medicine, Georgia Regents University, USA

## Abstract

**Background:**

Ischemic heart disease (IHD) is the major cause of death in patients with cardiovascular disease. Cardiac remodeling is a common pathological change following myocardial infarction (MI), and cardiomyocyte apoptosis plays a key role in this change. Transcription factor recombination signal-binding protein-J (RBP-J)-mediated Notch signaling pathway has been implicated in several inherited cardiovascular diseases, including aortic valve diseases, but whether the RBP-J-mediated Notch signaling pathway plays a role in cardiomyocyte apoptosis after MI is unclear.

**Method:**

We crossed RBP-J^fl/fl^ mice and Myh6-Cre/Esr1 transgenic mice to delete RBP-J in vivo and to partly inhibit the canonical Notch signaling pathway. MI was induced in mice by permanent ligation of the left anterior descending coronary artery followed by the knockout of RBP-J. Cardiac function and morphology were assessed by echocardiography and histological analysis 4 weeks after infarction. In addition, the expression and regulation of apoptosis-related molecules were examined by real time PCR and western blot.

**Results:**

RBP-J knockout decreased the survival rate and deteriorated post-MI remodeling and function in mice, and this effect was associated with increased cardiomyocyte apoptosis. The potential mechanisms might be related to the downregulated expression of bcl-2, upregulated expression of bax, and cleaved-caspase 3 to exacerbate cardiomyocyte apoptosis.

**Conclusion:**

These findings show that the RBP-J-mediated Notch signaling pathway in cardiomyocytes limits ventricular remodeling and improves cardiac function after MI. The RBP-J-mediated Notch signaling pathway has a protective role in cardiomyocyte apoptosis following cardiac injury.

## 1. Introduction

With the population growth and aging, the global burden of ischemic heart disease (IHD) is high [1, 2]. How to repair traumatic myocardium, reverse ventricle remodeling, and improve heart function after myocardial infarction (MI) are the main problems facing clinicians. IHD causes cardiomyocyte necrosis and apoptosis. Cardiomyocyte apoptosis, which is frequently seen in the border zone of ischemic tissue after MI, can lead to cardiomyocyte loss [3]. The regenerated cardiomyocytes are insufficient to replenish the lost myocytes, so in the border zone of ischemic tissue after MI, even relatively low levels of cardiomyocyte apoptosis have profound effects on cardiac remodeling and function [3, 4]. Bcl-2 and its family member bax are two upstream regulators of the mitochondrial apoptosis pathway [5]. Bcl-2 is known to be an antiapoptotic factor. Overexpressed bcl-2 protects cardiomyocyte viability and left ventricle function in IHD, whereas bax counteracts the antiapoptotic functions of bcl-2 [6].

A number of signaling pathways are involved in the regulation of cardiomyocyte apoptosis after MI. Notch signaling is an evolutionarily conserved pathway that governs cell fate specification. Activation of the Notch signaling pathway exhibits an improved hemodynamic function and reduced myocardial fibrosis after MI [7–9]. And there are many articles pointing that the Notch signaling pathway, especially activation Notch 1 receptor, protects cardiomyocyte apoptosis in several pathophysiological conditions, including hypoxia, ischemia/reperfusion, high glucose, burn, or lipopolysaccharide induced myocardial injury [9–12]. Notch signaling includes a RBP-J-dependent pathway called canonical Notch signaling and a RBP-J-independent pathway called noncanonical Notch signaling [13]. Most studies elucidated the cardioprotective effects of the Notch signaling pathway by activating or silencing individual Notch receptors. However, the role of RBP-J-mediated canonical Notch signaling pathway in cardiomyocyte apoptosis after MI is still unclear. Recombination signal-binding protein-J (RBP-J) is a DNA-binding protein and a key transcription factor downstream of receptor activation in the canonical Notch signaling pathway, but no Notch-independent functions of RBP-J have been described in the mammalian system [14, 15]. Therefore, knockout of RBP-J essentially generates a specific blockade of the canonical Notch signaling pathway. Moreover, in other tissues, RBP-J knockout inhibited cell proliferation and exhibited a reduction in the levels of bcl-2 and an upregulation of the expression of bax [16–18]. Our study supposes that, in adults, the RBP-J-mediated Notch signaling may reduce cardiomyocyte apoptosis after MI by regulating bcl-2/bax.

## 2. Methods

### 2.1. Mice and Animal Care

RBP-J^fl/fl^ mice and Myh6-Cre/Esr1 transgenic mice were kindly granted by Professor Li Hongliang (Animal Biosafety Level-III Laboratory, Wuhan University, Hubei, China). loxP sites were introduced on both sides of the RBP-J exons encoding its DNA-binding domain in RBP-J^fl/fl^ mice. To generate Myh6-RBP-J^fl/wt^ mice, the RBP-J^fl/fl^ mice were crossed with the Myh6-Cre/Esr1 transgenic mice with the use of the* Cre*-loxP system in which transgenic* Cre* expression is driven by the cardiac-specific mouse *α*-myosin heavy chain (*Myh6*) promoter [14, 19]. To induce Myh6-Cre-mediated recombination, Myh6-RBP-J^fl/wt^ mice were injected with 20 mg/kg Tamoxifen (Sigma Co., St. Louis, MO, USA), which was prepared in ethanol and diluted with corn oil; this mixture was administered by intraperitoneal injection once per day for 5 consecutive days at the age of 6-8 weeks [20]. Animal husbandry, experiments, and welfare were conducted in accordance with the Detailed Rules for the Administration of Animal Experiments for Medical Research Purposes issued by the Ministry of Health of China.

### 2.2. Murine MI Model

Myh6-RBP-J^fl/wt^ mice (*n* = 30) and RBP-J^fl/fl^ mice (*n* = 30) underwent MI surgery by permanent coronary ligation (*n* = 15 per group) or sham operation (*n* = 15 per group) 7-10 days after the intraperitoneal injection of Tamoxifen [21, 22]. In general, mice were anesthetized using Pentobarbital Sodium (50 mg/kg, i.p.) and mechanically ventilated. Left thoracotomy between the 3rd and 4th intercostal spaces was performed to expose the left ventricle. After removing the pericardium, the left coronary artery was ligated with an 8–0 silk suture approximately 2 mm below the edge of the left auricle. Sham-operated mice were subjected to a similar surgery without ligation. The chest and skin were closed with a 5-0 silk suture, and the animal was kept under mechanical ventilation until spontaneous breathing occurred and recovered on a heating pad [23].

### 2.3. Echocardiography

Echocardiography was performed with a MS400 probe on a Visual Sonics Vevo2100 small animal ultrasound scanner to assess cardiac function at day 0 and day 28. Mice were anesthetized (1.5% isoflurane and oxygen) and put in a supine position. Both two-dimensional and M-mode images were recorded. The left ventricular systolic dimension (LVDs), left ventricular diastolic dimension (LVDd), and septum and posterior wall thicknesses were measured. The left ventricular systolic volume (LV Vol s), left ventricular diastolic volume (LV Vol d), left ventricular ejection fraction (LVEF), and fractional shortening (FS) were computed from these measurements [24].

### 2.4. Histology

Hearts were harvested 28 days after operation. Hearts from the mice were isolated and fixed in 4% para-formaldehyde overnight. The hearts were then embedded in paraffin and sectioned at 3 *μ*m thickness. Hematoxylin and eosin (H&E) staining, Masson's trichrome staining, and terminal deoxynucleotidyl transferase dUTP nick-end labeling (TUNEL) staining were performed according to standard methods. Using H&E-stained sections, the cross-sectional area of cardiomyocytes was measured at the border zone. For Masson's trichrome-stained sections, photomicrographs were taken and collagen-positive areas were measured using ImageJ. TUNEL staining was performed to detect apoptosis. The nuclei of apoptotic cardiomyocytes were stained dark brown. Finally, the sections were counterstained with methyl green and then coverslipped. The sections were observed by light microscopy, and the apoptosis ratio was measured using ImageJ.

### 2.5. Western Blot

Left ventricular tissues were ground in liquid nitrogen. Nuclear protein and cytoplasmic protein extracts were made using a Nuclear Protein and Cytoplasmic Protein Extraction kit (Beyotime Biotechnology, Beijing, China) according to the manufacturer's instructions. The protein concentrations were determined with a BCA Protein Assay Kit (Beyotime Biotechnology, Beijing, China). Samples containing equal amounts of protein (20 *µ*g) were separated by 10% SDS-PAGE and transferred to a polyvinylidene difluoride (PVDF) membrane. After blocking for 1 h at room temperature, the membranes were probed with rabbit antibodies against RBP-J (1:500), bax (1:500), bcl-2 (1:500) (Santa Cruz Biotechnology, CA, USA), cleaved-caspase 3 (1:1000) (Abcam, Cambridge, UK), and *β*-actin mouse monoclonal antibody (1:1000) (Beyotime Biotechnology, Beijing, China) overnight at 4°C, followed by incubation with secondary horseradish peroxidase-conjugated anti-rabbit IgG antibody (1:1000) (Beyotime Biotechnology, Beijing, China) or horseradish peroxidase-conjugated anti-mouse IgG antibody (1:1000) (Beyotime Biotechnology, Beijing, China) for 1 h at room temperature. Proteins were detected by exposing the blots to X-ray film (Roche Applied Science).

### 2.6. Real Time Polymerase Chain Reaction

Real time PCR was performed to quantify and validate the specific target genes hairy and enhancer of split 1 (Hes1), the hes-related family bHLH transcription factor with YRPW motif 1 (Hey1), bax, and bcl-2 mRNA expression. *β*-actin was used as a control. Total RNA was isolated from left ventricular tissues using TRIzol reagent (Invitrogen, Carlsbad, CA, USA) according to the manufacturer's instructions. The concentration of RNA was measured using a NanoDrop ND-1000 spectrophotometer (Thermo Scientific, Waltham, MA, USA). One microgram of total RNA was reverse transcribed with Oligo (dT) primer using M-MLV reverse transcriptase (Invitrogen). Real time PCR was performed using SYBR Premix EX Taq (Takara) and the ABI PRISM 7500 real time PCR system. The PCR conditions involved a denaturation step (95°C for 10 sec), and amplification and quantification were repeated 40 times (95°C for 5 sec and 60°C for 30 sec, respectively). Sequences of the primers used in real time PCR were RBP-J (NM009035), forward: GAATGTACTTGTGCCTTTCTCAAGAAAG; reverse: CTGTAAGTTCAAGGATTGCTACGTCCCC; Hes1 (NM008235), forward: AGAGGCGAAGGGCAAG; reverse: AGGTGCTTCACAGTCATTT; Hey1 (NM010423), forward: GCATACGGCAGGAGGGAAA; reverse: CTGGGAAGCGTAGTTGTTGAGAT; Notch1 (NM008714), forward: TGCCAGGACCGTGACAACTC; reverse: CACAGGCACATTCGTAGCCATC; Bax (NM007527), forward: TCCACCAAGAAGCTGAGCGAG; reverse: GTCCAGCCCATGATGGTTCT; Bcl-2 (NM009741), forward: TTCTTTGAGTTCGGTGGGGTC; reverse: TGCATATTTGTTTGGGGCAGG; *β*-actin (M12481), forward: CATCCGTAAAGACCTCTATGCCAAC; reverse: ATGGAGCCACCGATCCACA. The relative target mRNA levels were determined using the 2^−ΔΔCt^ method.

### 2.7. Statistics

The statistical analysis was performed with the SPSS 19.0 program. The results were expressed as the means±SD. The comparisons between two groups were undertaken using an unpaired Student's t-test. The multiple comparisons were first analyzed by one-way ANOVA followed by Student-Newman-Keuls test to determine significance between groups. *P* < 0.05 was considered statistically significant.

## 3. Results

### 3.1. Knockout of the RBP-J Gene

**Figure 1:**
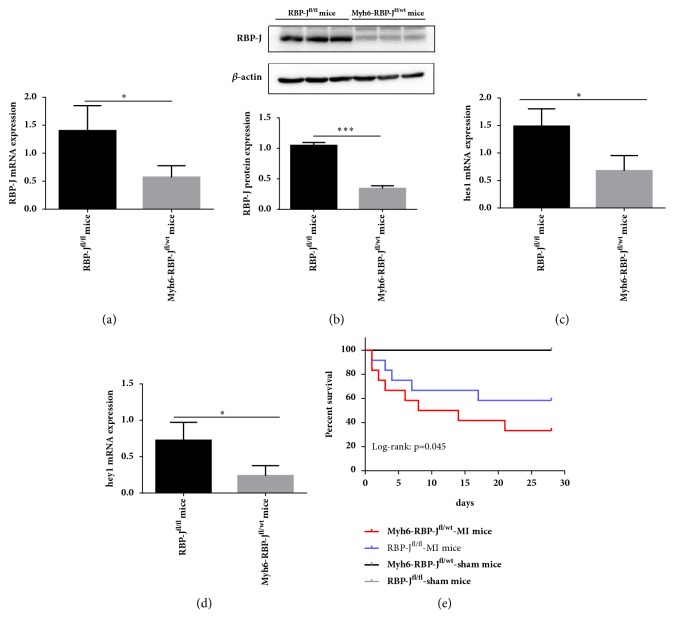
*Knockout of the RBP-J gene*. (a) Expression level of the RBP-J protein by western blot analysis. Significant differences were observed between Myh6-RBP-J^fl/wt^ mice and RBP-J^fl/fl^ mice (*n* = 3, ^*∗*^*P* < 0.05). (b&c) mRNA expression of Hes1 and Hey1 by real time PCR. Significant differences in the expression of Hes1 and Hey1 mRNA were observed in the myocardium of Myh6-RBP-J^fl/wt^ mice and RBP-J^fl/fl^ mice (*n* = 3, ^*∗*^*P* < 0.05). (d) Kaplan-Meier analysis showed a significantly lower survival rate in Myh6-RBP-J^fl/wt^-MI mice than in RBP-J^fl/fl^-MI mice (log-rank: *P* = 0.045).

We used Myh6-RBP-J^fl/wt^ mice as the experiment group and RBP-J^fl/fl^ mice as the control. Mice were injected with Tamoxifen for 5 days to induce Cre-mediated recombination and RBP-J knockout. The real time PCR and western blot analysis of the expression level of the RBP-J mRNA and protein in Myh6-RBP-J^fl/wt^ mice and RBP-J^fl/fl^ mice showed strong expression of the RBP-J mRNA and protein in the hearts of the RBP-J^fl/fl^ mice (Figures 1(a) and 1(b)). Here, we show that RBP-J knockout decreased the expression of Hes1 and Hey1 by real time PCR (Figures 1(c) and 1(d)). Significant differences in the expressions of Hes1 and Hey1 mRNA were observed in the myocardium of Myh6-RBP-J^fl/wt^ mice and RBP-J^fl/fl^ mice, which indicates that a specific haploid knockout of the RBP-J genes in the myocardium will also block the RBP-J-mediated Notch signaling pathway.

### 3.2. Myh6-RBP-*J*^*fl*/*wt*^ Mice Have a Lower Survival Rate following MI

After operation, Myh6-RBP-J^fl/wt^-MI mice have a higher mortality when compared with RBP-J^fl/fl^-MI mice or with Myh6-RBP-J^fl/wt^-sham mice and RBP-J^fl/fl^-sham mice. Postmortem examination indicated that the main cause of death was cardiac rupture and heart failure. The survival rate of Myh6-RBP-J^fl/wt^-MI mice was 33.3%, the survival rate of RBP-J^fl/fl^-MI mice was 58.3%, and the survival rate of Myh6-RBP-J^fl/wt^-sham mice and RBP-J^fl/fl^-sham mice was 100%. Kaplan-Meier analysis showed a significantly lower survival rate in Myh6-RBP-J^fl/wt^-MI mice than in RBP-J^fl/fl^-MI mice (log-rank: *P* = 0.045) (Figure 1(e)).

### 3.3. Deterioration of Cardiac Function in Myh6-RBP-*J*^*fl*/*wt*^ Mice following MI

To determine whether RBP-J knockout induces the deterioration of cardiac function following MI, echocardiography was performed at day 0 and day 28. We assessed LV Vol s, LV Vol d, LVEF, and FS in vivo as depicted in Table 1. LV Vol s was significantly increased in Myh6-RBP-J^fl/wt^-MI mice and in RBP-J^fl/fl^-MI mice, whereas LVEF and FS were decreased compared with Myh6-RBP-J^fl/wt^-sham mice and RBP-J^fl/fl^-sham mice. These results suggest that RBP-J knockout induces the deterioration of cardiac function following MI.

#### 3.3.1. Higher Collagen Density in the Infarct Area in the Myh6-RBP-*J*^*fl*/*wt*^ Mice following MI

**Figure 2:**
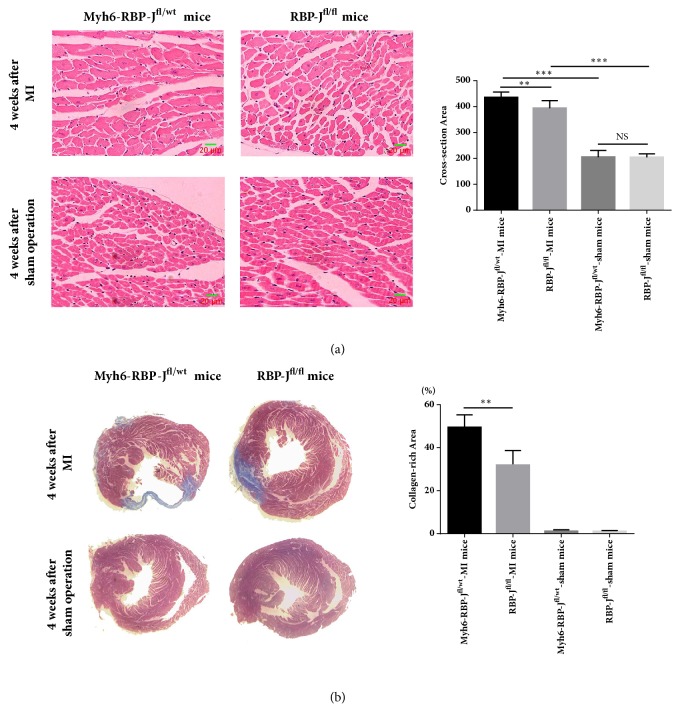
*Higher collagen density in the infarct area in the Myh6-RBP-J* ^*fl*/*wt*^ *mice following MI*. (a) 25 visual fields at the border zone were randomly selected from 4 mice for each group, and the cross-sectional area of cardiomyocytes was estimated (*n* = 4, ^*∗∗*^*P* < 0.01 and ^*∗∗∗*^*P* < 0.001). Data are shown as means ± SD. (b) 4 weeks after operation, the hearts of Myh6-RBP-J^fl/wt^-MI mice, RBP-J^fl/fl^-MI mice, Myh6-RBP-J^fl/wt^-sham mice, and RBP-J^fl/fl^-sham mice were sectioned and stained using Masson staining. Cardiac fibrosis was assessed by calculating the ratio of fibrotic scar (blue) average circumferences to left ventricular average inner circumferences (*n* = 4, ^*∗∗*^*P* < 0.01). Data are shown as means ± SD.

After 4 weeks, as shown by H&E staining, in both Myh6-RBP-J^fl/wt^-MI mice and RBP-J^fl/fl^-MI mice, the surviving myocardial cells were found in the border zones and arranged irregularly. The cross-sectional area of cardiomyocytes at the border zone was measured and demonstrated that Myh6-RBP-J^fl/wt^ mice with MI had an increased cross-sectional area of the border zone compared with RBP-J^fl/fl^-MI mice (Figure 2(a)). The analysis of Masson trichrome staining demonstrated that Myh6-RBP-J^fl/wt^ mice with MI had significantly increased fibrosis and collagen deposition compared with RBP-J^fl/fl^-MI mice (Figure 2(b)).

#### 3.3.2. RBP-J Knockout Increased Cardiomyocyte Apoptosis following MI

**Figure 3:**
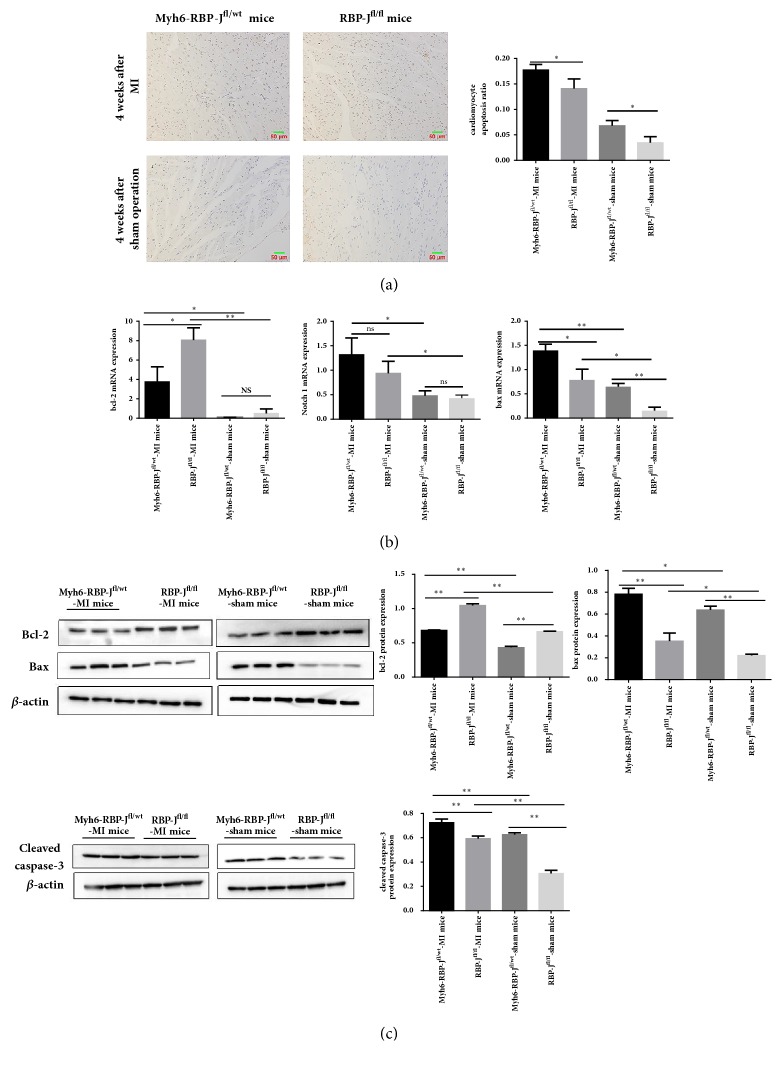
*RBP-J knockout increased cardiomyocyte apoptosis following MI*. (a) TUNEL staining showed that the number of apoptotic cells was increased in the border zone of ischemic heart tissue of Myh6-RBP-J^fl/wt^ mice compared with RBP-J^fl/fl^ mice with MI (original magnification: × 200, brown indicates TUNEL-positive nuclei and blue indicates nonapoptotic cells). The apoptosis rates of tissues in each group (Myh6-RBP-J^fl/wt^-MI mice, RBP-J^fl/fl^-MI mice and Myh6-RBP-J^fl/wt^-sham mice, and RBP-J^fl/fl^-sham mice) were detected (*n* = 6, ^*∗*^*P* < 0.05). Each value represents the mean ± SD. (b) Expression of bcl-2 and bax mRNA of different groups ((Myh6-RBP-J^fl/wt^-MI mice, RBP-J^fl/fl^-MI mice and Myh6-RBP-J^fl/wt^-sham mice, and RBP-J^fl/fl^-sham mice). (c) The protein expression level of bcl-2, bax, cleaved-caspase 3 by western blot analysis. The results are represented as mean ± SD (*n* = 6, ^*∗*^*P* < 0.05, ^*∗∗*^*P* < 0.01).

TUNEL staining was undertaken to analyze whether RBP-J knockout increased cardiomyocyte apoptosis. The results showed that in the border zone of ischemic heart tissue of Myh6-RBP-J^fl/wt^ mice compared with RBP-J^fl/fl^ mice after MI, the number of apoptotic cells was significantly increased (Figure 3(a)). We detected the apoptosis rates of tissues, and significant differences were observed between each group. We analyzed the expression of Notch 1, bax, and bcl-2 by real time PCR (Figure 3(b)) and the apoptosis proteins cleaved-caspase 3, bax, and bcl-2 by western blot (Figure 3(c)) to confirm the above results. We observed that the mRNA expression levels of Notch 1 and bax were significantly increased in Myh6-RBP-J^fl/wt^ mice compared with the RBP-J^fl/fl^ mice with MI, whereas bcl-2 expression was significantly decreased in Myh6-RBP-J^fl/wt^ mice compared with RBP-J^fl/fl^ mice following MI. The protein expression of cleaved-caspase 3 and bax was increased in Myh6-RBP-J^fl/wt^ mice compared with the RBP-J^fl/fl^ mice with MI, whereas bcl-2 protein expression had the same tendency as bcl-2 mRNA expression in Myh6-RBP-J^fl/wt^ mice compared with RBP-J^fl/fl^ mice following MI. These results indicated that in spite of activation of Notch 1 receptor, blockade of RBP-J-mediated Notch signaling pathway could deteriorate cardiomyocyte apoptosis induced by myocardial ischemia injury.

## 4. Discussion

This study produced that genetic blockade of RBP-J-mediated Notch signaling pathway receded the protective effect brought by activation of Notch 1 receptor. And this result was related to increased cardiomyocyte apoptosis in the RBP-J deficient mice after MI. The potential mechanism is involved in the modulation of an increased expression of bax and a decreased expression of bcl-2 after blockade of RBP-J-mediated Notch signaling pathway. These results indicate that endogenous RBP-J-mediated Notch signaling is critical for ischemia-induced myocardial injury, and this signaling pathway may serve as a therapeutic target.

Notch signaling is highly related to cardioprotective effects after myocardial injury. Previous studies found that Notch 1 was activated in myocardial injury [21]. And this result is similar to our study in which Notch 1 receptor was activated after MI. A recent study indicated that activation of Notch 1 limits the extent of ischemic damage, reduces myocardial fibrosis, and improves heart function [22]. In systemic Notch 1 deficient mice, myocardial infarction leads to the development of a larger myocardial infarct area and worsening of heart function than wild-type controls [25]. These results all manifested activation Notch 1 receptor has a protective effect after ischemia-induced myocardial injury. RBP-J is a key transcription factor downstream of receptor activation in the canonical Notch signaling pathway. In the present study, blockade of RBP-J-mediated Notch pathway led to deterioration of heart function, increased collagen deposition, and cardiomyocyte apoptosis after MI. These results indicated blockade of RBP-J-mediated Notch signaling pathway receded the protective effect brought by activation of Notch 1 receptor.

Apoptosis is uncommon in a normal heart but is a frequent process in cardiac remodeling, particularly in IHD [26–28]. And studies have shown that, in other tissues, the deletion of RBP-J is related to cell apoptosis [17, 29, 30]. In our study, we observed that blocking the canonical Notch signaling pathway by RBP-J knockout could increase cardiomyocyte apoptosis in the border zone of ischemic heart tissue following MI, which suggested that one cardioprotective effect brought by activation RBP-J-mediated Notch pathway is involved in the regulation of cardiomyocyte apoptosis.

With increasing incidence in recent years, the mitochondria are vulnerable to damage in response to ischemia/hypoxia, leading to excessive oxidative stress and apoptosis in cardiomyocytes [10, 31]. Bcl-2 and bax are two crucial mediators of the mitochondrial apoptotic pathway. Bcl-2 protein is a membrane protein and is mainly localized in the mitochondrial outer membrane. The mechanism of Bcl-2 inhibition of apoptosis may include (1) direct antioxidation; (2) inhibition of mitochondria release of proapoptotic proteins such as cytochrome C; (3) inhibition of cytotoxicity of bax; (4) inhibition of caspase activation [32, 33]. Meanwhile, bax initiates the mitochondrial apoptotic pathway by triggering the loss of mitochondrial membrane integrity, which releases cytochrome c from mitochondria into cytoplasm, activating caspase-9 and in turn, its downstream caspase-3, resulting in apoptosis [34, 35]. Previous studies have indicated that activation of Notch signaling pathway may have a role in protecting cardiomyocyte from apoptosis, and the potential mechanism may be related to the regulation of bcl-2 and bax [36], reduction of oxidative stress [11], and activation of other protective signaling pathway [29, 37]. Our findings show that the cardiomyocyte-specific blockade of RBP-J-mediated Notch signaling pathway after MI may involve the expression of pro- and antiapoptotic proteins, specifically bcl-2 and bax. And our research indicated that increased cardiomyocyte apoptosis induced by blockade of RBP-J-mediated Notch signaling pathway after MI may be related to the imbalance of bcl-2 and bax expression. Therefore, we thought RBP-J-mediated Notch signaling is capable of inhibiting the apoptosis-inducing effects of myocardial infarction, possibly through the regulation of bcl-2 family members. This effect was further reinforced by studies demonstrating that constitutively active Notch receptor or RBP-J modulates the expression of bcl-2 [16, 18].

In conclusion, we used genetic knockout of RBP-J-mediated Notch signaling pathway to demonstrate the roles of RBP-J-mediated Notch signaling in ischemia-induced myocardial injury. RBP-J-mediated Notch signaling also protects against ischemia-induced myocardial injury through the modulation of the expression of bax and bcl-2, which activates caspase 3 expression and leads to cardiomyocyte apoptosis. These findings suggest new therapeutic targets to limit ischemia-induced myocardial injury. However, the detailed underlying mechanism of the regulation of bcl-2 family members by the canonical Notch signaling requires further investigation in future studies.

## Figures and Tables

**Table 1:** The cardiac function at day 0 and day 28 following MI.

	Day 0		Day 28			
	Myh6-RBP-J^fl/wt^ mice	RBP-J^fl/fl^- mice	Myh6-RBP-J^fl/wt^-MI mice	RBP-J^fl/fl^-MI mice	Myh6-RBP-J^fl/wt^-sham mice	RBP-J^fl/fl^-sham mice
LVEF%	70.82 ± 0.40	72.97 ± 0.53	49.29 ± 4.00^*∗∗∗*^	56.20 ± 2.32	70.62 ± 0.51	72.29 ± 1.44
FS%	39.30 ± 0.23	41.17 ± 0.33	24.50 ± 2.35^*∗∗∗*^	28.56 ± 1.81	39.21 ± 2.48	40.64 ± 1.11
LVVol s	15.04 ± 1.40	14.64 ± 1.77	32.17 ± 8.00^*∗∗*^	22.92 ± 6.62	16.22 ± 2.62	15.55 ± 2.33
LVVol d	51.51 ± 4.13	54.10 ± 5.66	63.10 ± 12.18	61.49 ± 13.11	55.11 ± 7.95	55.93 ± 5.90

At day 0, there was no significant difference between Myh6-RBP-J^fl/wt^ mice and RBP-J^fl/fl^ mice in cardiac function. At day 28, LVEF and FS were smaller, while LV Vol s was greater in Myh6-RBP-J^fl/wt^-MI mice compared with RBP-J^fl/fl^-MI mice. And there was no significant difference between Myh6-RBP-J^fl/wt^-MI mice and RBP-J^fl/fl^-MI mice in LV Vol d. Data are shown as mean±SD. *n* = 6 for RBP-J^fl/fl^ mice with sham operation, 6 for Myh6-RBP-J^fl/wt^ mice with sham operation, 12 for RBP-J^fl/fl^ with MI, and 12 for Myh6-RBP-J^fl/wt^ mice with MI. ^*∗∗*^*P* < 0.01 versus RBP-J^fl/fl^ with MI in LV Vol s. And ^*∗∗∗*^*P* < 0.001 versus RBP-J^fl/fl^ with MI in LVEF and FS by Student's t-test.

## Data Availability

The data used to support the findings of this study are available from the corresponding author upon request.
